# Physician Response Time When Communicating With Patients Over the Internet

**DOI:** 10.2196/jmir.1583

**Published:** 2011-11-01

**Authors:** Per Egil Kummervold, Jan-Are K Johnsen

**Affiliations:** ^1^Norwegian Centre for Integrated Care and TelemedicineUniversity Hospital of North Norway HFTromsøNorway; ^2^Department of Clinical MedicineFaculty of Health SciencesUniversity of TromsøTromsøNorway

**Keywords:** Electronic mail, Internet, patients, physicians, patient communication, health communication

## Abstract

**Background:**

Patients want to use electronic communication to access health services more easily. Health authorities in several countries see this as a way to improve health care. Physicians appear to have conflicting opinions regarding the suitability of electronic communication in clinical settings.

**Objectives:**

The aim of our study was to measure how long it actually takes physicians to answer questions from patients through an electronic communication channel, and whether some of the questions are especially time consuming.

**Methods:**

We monitored electronic patient–physician communication. A total of 1113 messages from 14 participating physicians from 7 medical offices were analyzed. The length of questions and answers, and the time physicians spent answering the questions were recorded and analyzed.

**Results:**

Physicians spent an average of 2.3 minutes (median 2 minutes) answering questions from patients. The patients’ questions had an average length of 507.1 characters (95% CI 487.4–526.9, SD 336.2), while physicians’ answers averaged 119.9 characters (95% CI 189.8–210.0, SD 172.6). The results show that the influence of patient question length on time spent responding was negligible. For the shortest 25% of the questions the answer time was 2.1 minutes (95% CI 1.9–2.3), while it was 2.4 minutes (95% CI 2.2–2.7) for the longest 25%. Even extremely long questions had a minimal impact on the time spent answering them. A threefold increase in question length from patients resulted in only an 18% increase in physician response time.

**Conclusions:**

The study shows the potential clinical usefulness of electronic communication between patients and health care services by demonstrating the potential for saving time.

## Introduction

While the majority of the European population are using the Internet for health purposes, only 1 in 10 Internet users communicate directly with their physician over the Internet. However, this number is rising, increasing from about 5% in 2005 to 9.7% in 2007 [1]. This increase appears to be driven both by patients wanting easier access to health services and by health authorities wanting to make health care more efficient [2].

Among physicians there appear to be conflicting opinions regarding the usefulness of electronic communication in clinical settings. Patt and colleagues [3] reported that some physicians saw email as more convenient, more flexible, and time saving. In contrast, others felt that email could become an added burden, especially if the physician was solely responsible for handling the contact. Also, physicians have expressed concerns that patients’ messages might be inappropriate and inefficient [4]. In sum, physicians’ negative perceptions of email contact appear linked to the concern that answering questions from patients will take too much time, and in particular that answering long and complex questions will consume a disproportionate amount of time.

In Norway, purpose-written applications are used for patient–physician communication, since ordinary email does not meet the required security level set by the Norwegian Data Inspectorate. Apart from the user having to log on using a password and one-time codes, the systems provide the same functionality as an email system. From the physicians’ point of view, they do, however, integrate more tightly with the electronic patient record. Evidence appears to support that purpose-written applications can be at least as cost effective in large-scale use as email [5]. Also, there is evidence that electronic communication is replacing some traditional inquiries, including visits [6] and telephone calls [7,8], and in general patients hold a positive view of electronic access to health care providers[7].

This study aimed to measure how long it actually takes physicians to answer electronic questions from patients, and whether some of the questions are especially time consuming. Two main hypotheses were posed:

A: The length of questions from patients predicts the time physicians spend answering.

B: The longest questions consume an unreasonably large amount of physicians’ time resources.

It is obvious that how long a time a physician uses to compose an answer is correlated with the number of characters he or she is typing. However, the strength of the correlation should be investigated, especially in relation to how long the message from the patient is.

## Methods

We asked the 2 suppliers of secure patient communication systems in Norway, Visma Unique [9] and DIPS [10], to provide us with a list of the offices that used the systems actively, and where the systems were integrated with the electronic patient record system. At the time of the study, these 2 systems were the only ones in use in Norway that enabled secure patient–physician communication. From a list of 13 offices, 9 were willing to participate in the study. Due to technical issues, the data from 2 of these offices were inaccessible, leaving us with 7 offices included in the study.

A program logging the time physicians spent answering and the length of the patient questions was installed at the offices included in the study. Time was logged by automatically recording how long the physician took from opening the patient question to sending the answer. In addition, the program recorded the length of the question and of the answer. Prior to sending the answer, the physician was presented with a dialog box indicating the time that had elapsed. This time estimate could then be adjusted if the physician felt this was inaccurate. Both additive and subtractive adjustments could be made. For instance, subtractive adjustments could be made if the physician was interrupted while typing, and additive adjustments could be made if the physician had used more time composing the answer than was recorded by the system. The adjusted time had to be given as an integer. Unadjusted time was therefore also rounded to the closest positive integer, giving a minimum answer time of 1 minute. A total of 380 adjustments were made.

The study ran for 1 year, starting December 2005. A total of 1321 messages were recorded in the period. Physicians sending fewer than 10 messages (n = 1) and physicians not completing the task of returning the data (n = 3) were excluded. Office personnel were not included. This resulted in 14 participating physicians (3 female) and a total of 1113 messages. The physicians had on average worked 15.7 years (range 3–30 years) and had an average patient load of 1441 (range 1100–2300 patients).

The target patient population was all those using primary health services. Earlier studies have shown that young, well-educated persons are overrepresented in using electronic health services [11].

The Regional Committee for Medical and Health Research Ethics approved the study. Hypotheses were investigated by descriptive statistics and linear regression analysis. Data were analyzed using SPSS version 18.0 (IBM Corporation, Somers, NY, USA).

## Results

Questions from patients averaged 507.1 (95% CI 487.4–526.9, SD 336.2) characters in length, while the physicians’ answers averaged 119.9 (95% CI 189.8–210.0, SD 172.6) characters. Physicians spent an average of 2.3 (SD 2.0) minutes answering questions; 17 (1.5%) of the questions took more than 10 minutes to answer, while 125 (11.2%) of the questions took between 5 and 10 minutes to answer. Table 1 summarizes the descriptive statistics and Table 2 shows the time the participating physicians spent answering patient questions.

**Table 1:** Descriptive statistics of question length for patients and physicians

	Minimum	Maximum	Mean	SD
Patient question length (number of characters)	100	3315	507.1	336.2
Physician answer length (number of characters)	14	1634	119.9	172.6

**Table 2:** Response time of participating physicians

Physician ID	Number of questions answered	Mean (minutes)	Median (minutes)		
			25%	50%	75%
A	68	2.7	1	2	3
B	187	2.1	1	2	2
C	123	3.2	1	2	5
D	24	2.5	2	2	3
E	18	1.5	1	1	2
F	20	1.8	1	1	1
G	74	3.0	2	3	4
H	46	2.8	1	2	3
I	39	1.2	1	1	1
J	12	1.2	1	1	1
K	79	1.7	1	1	2
L	218	2.1	1	1	3
M	82	1.8	1	1	2
N	123	2.0	1	1	2
Total	1113	2.3	1	2	3

We expected that the length of the patients’ questions would predict response time (hypothesis A). The hypothesis was investigated through regression analyses. Two models were tested. The first model included only the length of the patients’ questions. While the model significantly explained variance (*P* = 0.007), the effect size was small (beta = .08) and the overall fit of the model was very low (*R* *2* = .01). The second model included also the length of the physicians’ answers (Table 3) and showed better fit (*R* *2* = .26). The results indicate that the influence of patient question length on response time is negligible (beta = –.05, *P* = .05) compared with the length of the physician’s answer (beta = .53, *P* < .001).

**Table 3:** Summary of regression analysis for patient question length and physician answer length predicting response time (minutes)

Model	B	SE	Beta	*t*	*P* value
(Constant)	1.20	.10		12.02	<.001
Patient question length	.00	.00	–.05	–1.94	.05
Physician answer length	.01	.00	.53	19.75	<.001

Patient questions were categorized based on their length. Patient questions were divided into quartiles each containing 25% of the messages (Table 4). This confirmed that for most of the questions, the effect of question length on answer time was negligible. The answer time was 2.1 minutes (95% CI 1.9–2.3) for the shortest 25% of questions and 2.4 minutes (95% CI 2.2–2.7) for the longest 25%.

**Table 4:** Time physicians spent answering patients’ questions by question length

Patient question length		Number of answers	Physician answer time in minutes (95% CI)
Quartile	Number of characters		
1	0–308	278	2.1 (1.9–2.3)
2	309–398	280	2.3 (2.1–2.5)
3	399–594	277	2.2 (1.9–2.5)
4	595–3315	278	2.4 (2.2–2.7)

Hypothesis B states that the longest questions would consume an unreasonably large amount of physicians’ time resources. These questions were defined as being the top 10% of questions (110 questions) with regard to length (>916 characters) (see Table 5).

**Table 5:** Time physicians spent answering the longest 10% of patients’ questions

	Number of questions	Patient question (mean number of characters)	Physician answer time (minutes)
Shortest 90% of questions (≤916 characters)	1003	420	2.2
Longest 10% of questions (>916 characters)	110	1300	2.6

As shown in Table 5, the 10% longest patient questions were approximately 3 times the length of shorter questions (420 characters versus 1300 characters). However, the physicians spent on average 18% more time answering the 10% longest questions (2.6 minutes compared with 2.2 minutes). These results were not in favor of hypothesis B.

## Discussion

The results give mixed support to the hypotheses. As expected, the length of patients’ questions predicted answer time, but the analysis also shows that the predictive value is negligible compared with the length of the physicians’ answers. We did not find conclusive support for the hypothesis that very long patient messages should have a large effect on physician answer length and answering time. Instead, we observed a modest increase in physician answer length and only a marginal increase in answering time related to extremely long patient questions.

As noted, one of the main reasons physicians are skeptical about electronic communication is the potential for increased workload [12]; for instance, physicians might fear that patients would overuse it or that responding to questions would be time consuming. The results of the current study show, however, that these specific concerns might be unfounded. While it does take extra time to read long questions from the patient, this does not have large effects on the total time used by physicians to answer patient inquiries. In fact, a threefold (300%) increase in patient question length resulted in only an 18% increase in physician response time. Even though the average numbers may support the effectiveness of an electronic communication system, and other studies indicate that responses to email messages do not take more time than responses to nonelectronic patient messages [13], one may still question whether extreme cases will jeopardize these effects in a real-life office setting. Based on the current results these concerns appear unfounded. The average physician response time to a patient message was 2.3 minutes using the systems described in this study. Only 1.5% of questions took more than 10 minutes to answer. When compared against the average consultation time in Norwegian general practice (15–20 minutes) [14], even these unusual cases will have to be regarded as time saving, if the electronic messages substitute patients’ office visits [6]. It is, however, unlikely that electronic messages can be a substitute for office visits in a one-to-one relationship. Other studies have shown that electronic messages can replace phone calls [7,8], and it is very likely that electronic messaging will find relevance as a supplement to personal encounters, for instance by recommendation of ethical guidelines [15].

Results from other investigations indicate that patients are willing to adapt to guidelines regarding the focus and content of messages [4], which should help to minimize the potential problem of lengthy patient questions. Obviously, the time-saving potential is highly dependent on electronic messages substituting for patient office visits [6].

### Limitations

This study included a considerable proportion of Norwegian physicians using electronic patient communication at the time the study was performed. It is not self-evident that the result would be valid for all physicians using similar services. An alternative approach would be to select a random sample of all physicians using electronic communication. At the time of the study, only a few Norwegian physicians were offering electronic communication services. A random sample could therefore be biased toward physicians being positive to electronic communication. In some countries, for instance Denmark [1], it has become mandatory for physicians to offer electronic communication services. In such contexts, a similar study based on random selection would be feasible.

The current study does not involve analysis of the content of the messages. The main challenge in doing this would be that it would require written consent from every patient. However, a prior study in similar populations has shown that only a small proportion of these messages are used for simple administrative purposes such as scheduling [11]. Instead, the majority of the patient messages are concerned with health-related questions, and requesting prescriptions, test results, and documentation for medical leave.

The average time spent answering messages might be influenced by factors such as workload and reimbursement policies. This limits the external validity of the current results. However, the investigated relationships between variables (eg, that the length of patients’ messages had limited impact on the answer time), rather than their absolute values, are much more likely to also be valid in other cultural contexts.

### Conclusions

Studies have demonstrated how email and electronic messaging systems can be used to promote balanced and patient-centered communication [16], in support of clinical decision making [17-19]. We believe the results reported here further extend the clinical usefulness of electronic communication between patients and health care providers by demonstrating the potential for saving time.
